# The Influence of the Type of Lime on the Hygric Behaviour and Bio-Receptivity of Hemp Lime Composites Used for Rendering Applications in Sustainable New Construction and Repair Works

**DOI:** 10.1371/journal.pone.0125520

**Published:** 2015-05-27

**Authors:** Anna Arizzi, Monika Brümmer, Inés Martín-Sanchez, Giuseppe Cultrone, Heather Viles

**Affiliations:** 1 School of Geography and the Environment, University of Oxford, Dyson Perrins Building, South Parks Road, Oxford, OX1 3QY, United Kingdom; 2 CANNABRIC, Cañada Ojeda, 8 E-18500 Guadíx, Granada, Spain; 3 Departamento de Microbiología, Universidad de Granada, Avda. Fuentenueva s/n, 18002, Granada, Spain; 4 Departamento de Mineralogía y Petrología, Universidad de Granada, Avda. Fuentenueva s/n, 18002, Granada, Spain; Institute for Materials Science, GERMANY

## Abstract

The benefits of using sustainable building materials are linked not only to the adoption of manufacturing processes that entail reduced pollution, CO_2_ emissions and energy consumption, but also to the onset of improved performance in the building. In particular, hemp-lime composite shows low shrinkage and high thermal and acoustic insulating properties. However, this material also shows a great ability to absorb water, an aspect that can turn out to be negative for the long-term durability of the building. For this reason, the hygric properties of hemp-based composites need to be studied to ensure the correct use of this material in construction and repair works. The water absorption, drying and transpirability of hemp composites made with aerial (in the form of dry powder and putty) and hydraulic limes were investigated here and related to the microbial growth induced by the water movements within the material. Results show that hemp-natural hydraulic lime mixes exhibit the highest transpirability and drying rate, the lowest water absorption by immersion and capillary uptake and the least intense microbial attack and chromatic change. A microscopical study of the hemp shives also related their great ability to absorb water to the near-irreversible swelling of their structure under dry-wet conditions.

## Introduction

Hemp-based composite (also called *hemp concrete* or *hemp lime*) is a lightweight concrete produced by mixing hemp shives (i.e. inner woody part of the stem, also called shives) with a binder. Since its production entails the use of a natural and renewable resource (the hemp plant), low carbon and pollutant emissions during the manufacturing process, low net waste at the construction site, increased energy efficiency in the building and improved quality of indoor air, hemp-based composite is being increasingly used as sustainable building material, as a valid and efficient alternative to traditional construction.

In particular, hemp-based composites are characterized by a very low bulk density and high porosity when hardened, exhibit a very low thermal conductivity (λ = 0.1–0.2 W/m·K), a high acoustical absorption factor compared to traditional concrete and, finally, a high fire resistance [1], without the need to use fire-retardant additives.

Due to these unique characteristics, many applications are possible for hemp-based composites, such as: insulating filling materials and panels for walls and roofs, masonry blocks and renders. Notwithstanding, because of the very low mechanical resistance to compressive and flexural tensions achieved with hemp-lime mixes, their use is usually not recommended for structural purposes [2,3]. Moreover, the elaboration of loadbearing hemp-lime composites (i.e. mortars with structural purposes) implies using a material with higher density and lower thermal conductivity, which is the reason why only non-loadbearing hemp composites are common in countries with cold climates. In relation to the poor mechanical resistance of this type of material, many authors have highlighted the need for pre-treating (e.g. with alkali) different plant shives to increase their surface roughness thus improving the adhesion of polymers or binder particles and, consequently, the mechanical resistance of the composite materials [4–6]. However, other studies also demonstrate that alkaline treatment of hemp shives causes an increase in water sorption by capillary uptake and water retention ability of the fibres [7,8]. This treatment, indeed, induces swelling of the crystalline structure of cellulose, during which the hydrogen bonds between the hydroxyl groups are broken, which allows the formation of other hydrogen bonds between the hydroxyl groups and water molecules [9].

Besides the high water absorption of hemp shives (they can absorb 300–400 times their weight in water [10,11]), they also show a good transpirability [1]. This characteristic encourages the use of hemp shives especially in rendering mortars, since they might lead to a reduction of moisture in the interior of a building.

In summary, the use of hemp-based composites for rendering applications presents several advantages, such as the decrease of the material density when hemp shives are used in the place of a stone aggregate (lightweight rendering mortar); improvement of the water retention properties of the render and consequent reduction of shrinkage; increase of the material flexibility and consequent decrease of the tensions between the render and the materials of the wall; and development of a healthier environment in the interior of the building.

### The use of hemp lime composite as repair rendering material

The characteristics above detailed justify the use of hemp lime renders not only in new construction works but also in repair interventions, especially focused on improving the insulation and hygrothermal performance (i.e. rendering or plastering applications) of historic buildings (e.g. rammed earth, adobe and cob constructions [12]). However, taking into account both the physical characteristics of the shives (high porosity and specific surface area [10]) and the chemical interactions taking place between water and cellulose [9,13], which are also responsible for the water sorption properties of hemp shives, it is of fundamental importance to assess the hygric behaviour of a hemp-based composite when it is used as repair material. In fact, although great water absorption and swelling ability is required in many industrial processes in which hemp is involved (such as dyeing, bleaching of tissues), this is not desirable in building and repair application.

One of the requirements that a repair material must meet to improve the durability of the building is, in fact, a limited water absorption capacity [14,15]. The presence and movement of water within the pore system of a building material, under suitable environmental conditions, enable degradation processes [16,17], such as freezing-thawing phenomena, salt crystallization and microorganism growth, which reduce the durability of the material and create an unhealthy environment.

### The choice of the binder

The hygric behaviour of a composite building material obviously depends on the components and dosages used, as well as on the preparation procedure followed (e.g. mixing, casting, tamping). Regarding the choice of the correct binder, mixing hemp with lime seems to be the most convenient solution for producing carbon-negative hemp-based construction materials (both hemp and lime are efficient CO_2_-sequesters). However, hemp lime mixes are often produced with a formulated lime-based binder instead of lime on its own [18]. According to the European Standard BS 459–1 [19] a formulated lime (FL) can be made with the following components in variable proportions: air-hardening lime (CL), natural hydraulic lime (NHL), cement (which can contain clinker, natural pozzolan, limestone, granulated blast furnace slag, plus minor constituents, such as calcium sulphate and silica fume, and organic or mineral additives [20]). Two of the most common formulated binders available on the market for the production of hemp lime composites are Tradical and LimeGreen, which are both a combination of lime, cement and other additives [18]. Although in the last decade the quantity of CO_2_ emitted due to cement manufacturing has decreased [11], as cement is increasingly manufactured from recycled materials [18], its production still embodies higher consumption of combustible and therefore emission of more CO_2_ and pollutant gases, with respect to lime [11]. Furthermore, cement is not a compatible and suitable binder for conservation works on historic buildings, as demonstrated by the irreversible damages and acceleration of the decay processes that its application has caused to the architectural heritage [21].

For these main reasons, in this study we have avoided the use of formulated binders and preferred instead to use three limes commonly used in restoration works: natural hydraulic lime and air-hardening lime in the form of dry hydrated powder and putty.

The water sorption and desorption properties and the transpirability (i.e. permeability to water vapour) of the three types of hemp mixes were assessed by means of standardised and non-standardised hygric tests and then were related to the water absorption ability of the hemp shives, studied at microscopic scale. Furthermore, the bio-decay of these materials under wet-dry conditions was examined. The main objective of this work was to assess the influence of the type of lime on the hygric behaviour and bio-receptivity in the presence of water of hemp lime mixes, with the final aim to indicate the most suitable lime to be used in combination with hemp for rendering applications in sustainable new construction and repair works.

## Materials and Methods

### Materials

Three types of lime were selected to produce the hemp composites: a hydrated lime in the form of dry powder (CL90-S [19]) produced by ANCASA (Seville, Spain); a hydrated lime in the form of putty stored under water for 2 years (CL90-S PL [19]), produced by ComCal (Barcelona, Spain); and a natural hydraulic lime (NHL3.5 [19]) produced by Socli, Italcementi Group (Izaourt, France).

The hemp, whose commercial name is Cannhabitat, is produced by AgroFibre, Euralis (Cazeres, France) and was supplied by the Cannabric enterprise (Guadix, Granada, Spain). Hemp plants are usually collected at the end of summer and dried on site. The hemp shiv (i.e. the woody inner core of the stem), which is the part of the plant used for construction purposes, is cut to elongated and flat particles of size ranging from 2 to 25 mm.

A microbiological study was carried out on the hemp shives, although their technical sheet implied they were exempt from pathogen microorganisms (for further details see www.cannabric.com). For this study, 0.25 g of hemp shives were placed in 500 μL of sterile deionised water; samples were vigorously agitated by means of a mechanical shaker and then inoculated onto plates containing Trypticase soy agar (TSA, Scharlau Chemie S.A., Barcelona, Spain) and Sabouraud chloramphenicol agar (Scharlau) media (100 μL of the suspension obtained per plate) and incubated at 28°C for one week. During this period, colonies exhibiting different morphology and appearance were transferred to new culture plates of TSA medium for bacteria and potato dextrose agar (PDA) for fungi, to obtain pure strains. Phenotypic characterization of isolated microorganisms was performed by observation of macroscopic features such as colour, shape and texture of the colonies that appeared in the culture media. Hyphae, sporangia and spores of fungi have been visualized by staining with lactophenol blue. Bacteria were identified by Gram staining. Observation of the samples was performed with a Leitz Dialux 22 optical microscope with objectives of 60X/100X. Images were obtained with an Olympus Camedia C-5060 camera coupled to the microscope.

### Hemp lime composite production

Hemp lime mixes were produced by hand and so did not follow the specifications of the European standard for mortars (e.g. the order of mixing components, the use of an automatic mixer, the establishment of the optimum amount of water by means of a flow test and the curing conditions and time). Modifications to these specifications were made to improve the technical quality of the final material.

The lime:hemp:water dosage by volume was 3:5:2.5 for the limes in the form of a powder (CL90S and NHL 3.5). In the case of the lime putty (CL90S PL) the same volume of lime and hemp but a lower amount of water were used. The difference in the water content was calculated considering the differences in the bulk density between the powdered (~ 540 g/L) and the putty (~ 1205 g/L) lime.

The steps of hemp lime preparation were:

1. the hemp shives were mixed with half of the amount of water;
2. the lime was added and mixed with the hemp shives;
3. the rest of the water was added and the components mixed.

**Fig 1:**
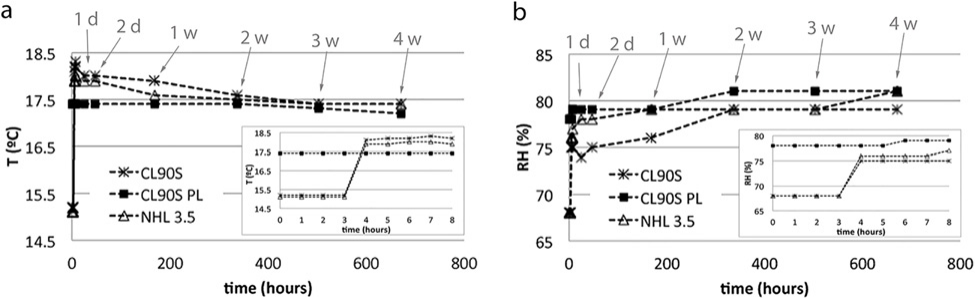
Environmental conditions measured during the first month of curing hemp lime composites. T (in °C, a) and RH (in %, b) and represented as a function of time (t, in hours). The insets show T and RH conditions during the first 8 hours. Arrows indicate the time in days (d) or weeks (w).

Mixing was performed in a drum with vertical axis and rotating arms (500 L, 5800 W). Hemp composites were cured in the Cannabric factory base for three months under average conditions of T = 17°C and RH = 75%. As shown in Fig 1, the environmental curing conditions were slightly different for the three mix types, since they were not produced at the same time but in succession.

### Study of the hygric behaviour of hemp lime composites

Water loss of the hemp lime composites due to drying was evaluated by measuring the weight of samples every hour for the first 8 hours, then at 24 and 48 hours and then once a week for the following 4 weeks, after the preparation of the materials. Weight variations were measured on 12 samples per mix type. During the first week samples were in the mould, therefore three samples per mould were weighed at the same time.

The water vapour permeability (K_v_, in gm^-2^h^-1^) was determined on three samples (1.5×4×4 cm) per mix type, following the same experimental procedure as in Arizzi and Cultrone [22]. This test was performed for 5 days in total, under controlled conditions (T = 25°C; RH = 40±5%).

Absorption and drying rates were determined on three samples (4×4×4 cm) per mix type by measuring the changes in the weight of samples over time. The absorption coefficient (C_a_, in g min^-1/2^ [23]) was determined as the slope of the curve representing the weight increase (ΔM/M, in %) as a function of the square root of time (in min), 1 minute after the beginning of the test (not 4 minutes as the standard specifies, because mortars absorb water faster than rocks, especially hemp-based ones). The drying index (I_d_) was measured according to the NORMAL 29–88 standard [24].

Capillary rise tests were performed on three samples of 4×4×16 cm, following the UNI-EN 1925 standard [25]. The imbibition coefficient (C_u_) was determined as the slope of the curve representing the weight uptake per surface area (ΔM/MS, in %) as a function of the square root of time (in min), 1 minute after the beginning of the test.

Before the hygric tests, samples were oven-dried at 90°C for 8 hours.

### Study of the changes in hemp lime composites after the hygric tests

The surface of samples before and after the capillary test was observed by means of a digital videomicroscope Leica DVM2000. A microbiological study was performed on each sample after the water capillary uptake test. Two types of samples were collected to characterize the microbial community present in the hemp lime mixes: swab samples (sterilized by ethylene oxide and individually wrapped in peel-pack) deemed suitable for isolations in culture media (Class IIa) (Eurotubo, Deltalab, Rubí, Spain) and adhesive tape samples, both from areas showing colour changes. The microbiology study of hemp lime mixes was performed following the same experimental procedure as the one described above for the study of the hemp shives.

Chromatic differences between the different mix types, as well as differences before and after water absorption in each composite, were detected by means of a portable Konica-Minolta CM-700d spectrophotometer. The CIELab system was chosen and L* (lightness, from 0 to 100) and a* and b* (chromatism, from -60 to +60) parameters calculated by means of reflectance values [26]. The measurement conditions were as follows: measurement area of 8 mm, D65 standard illuminant and observer 10° with modes SCI/SCE and a wavelength range from 400 nm to 700 nm with a wavelength interval of 10 nm. Ten measurements per mix type were performed. The overall colour difference (ΔE) of the three mixes before and after the water absorption-drying and the capillary uptake tests was determined as follows: ΔE = $\sqrt{\left({\left(L_{1}^{*}-L_{2}^{*}\right)}^{2}+{\left(a_{1}^{*}-a_{2}^{*}\right)}^{2}+{\left(b_{1}^{*}-b_{2}^{*}\right)}^{2}\right)}$, where L_1_*, a_1_* and b_1_* are respectively the lightness and the chromatic coordinates of the untreated samples and L_1_*, a_1_* and b_1_* are those of samples after the water absorption-drying or the capillary tests. A two-way analysis of variance (ANOVA) was carried out to investigate the influence of two different factors (type of lime and test) on the chromatic variations of hemp lime composites. For this analysis, “Mix” and “Test” were two fixed factors with three levels each: CL90S, CL90S PL, NHL 3.5 and before, after water absorption-drying, after capillary uptake, respectively. Three separate analyses were carried out with L*, a* and b* values (n = 10) as dependent data.

### Study of the water absorption and drying of hemp shives at the micro-scale

To better understand the water absorption behaviour of the hemp shives, they were submitted to cycles of hydration and dehydration in a Philips Quanta 400 environmental scanning electron microscope (ESEM), which worked at a fixed temperature of 2°C. Small pieces of hemp shives (~5 mm) were placed in the chamber, without being dried or sputter coated. Before observation, the microscope chamber was purged 5 times at a range of pressures between 2.5 and 5.5 torr, which correspond to relative humidity of approximately 50% and 100%, respectively. Once equilibrium was achieved, the hydration dehydration cycles were performed at a range of pressure from 3.5 to 6 torr, which simulated a relative humidity range between 70% and over 100%. At around 5 torr, hemp shives were soaked in water and, at the highest pressure, water condensed on their surface. Relative humidity was never decreased below 70% to avoid modifying the structure of the shives.

## Results and Discussion

### Weight loss during drying

**Fig 2:**
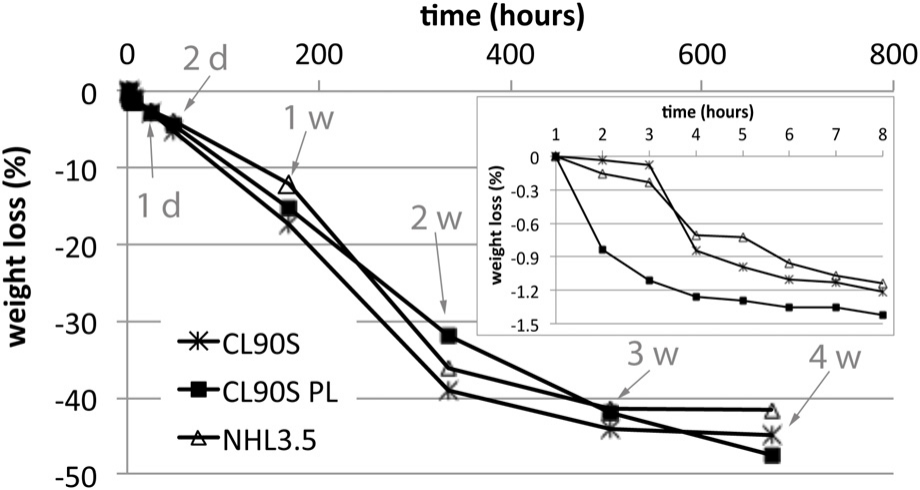
Weight loss measured during the first month of curing hemp lime composites. The weight (in %) of the three hemp lime mixes is represented as a function of time (in hours). The inset shows the weight loss during the first 8 hours. Arrows indicate the time in days (d) or weeks (w).

Fig 2 shows the weight loss of the three different hemp lime mixes, as a result of drying during the first month of curing. It is important to notice that the environmental conditions of T and RH were stable only during the curing of CL90S PL samples, whilst the other two mixes (made with CL90S and NHL 3.5) were exposed to slight variations of these conditions (Fig 1) that undoubtedly influenced their drying behaviour, especially during the first 3 hours. During this period, indeed, CL90S and NHL 3.5 samples were exposed to 2.5°C less of temperature and 10% less of relative humidity than CL90S PL samples. This led to slightly less weight loss in the CL90S and NHL 3.5 composites (~0.1%) compared to the CL90S PL one (1%, inset in Fig 2), at the beginning of the drying process. From the 4^th^ hour onwards, temperature and relative humidity reached similar average values for all hemp lime mixes and this made their drying behaviour more comparable over the following days. From the 4^th^ to the 8^th^ hour of curing (inset in Fig 2), the three composites lost water at similar speed (the curve shows a similar slope) but their drying rate changed again after one week of curing (corresponding to 168 hours). During the second week, in fact, CL90S and NHL 3.5 samples lost almost 23% of their initial weight against the 16% lost by CL90S PL samples. After this period, the weight of the former hemp lime composites started to stabilise (between the 3^rd^ and the 4^th^ week the weight loss in these mortars was less than 1%), whilst CL90S PL samples continued losing water after one month (5% of water was still lost during the last week of measurement).

It is worth noticing that, in spite of the incomparable weight variations at the beginning of the drying process, the type of lime does seem to influence in some way the water loss rate of hemp lime composites, in contrast with the findings of Colinart et al. [3]. Weight loss curves indicate that although the CL90S PL mix loses more water (because of its higher initial water content) its drying is slower and more linear over time. This behaviour is more desirable in a render since it reduces tensions in the material due to shrinkage (i.e. volume reduction due to rapid water loss during drying). The lowest weight loss, however, was registered in NHL 3.5 samples.

### Water vapour permeability

**Fig 3:**
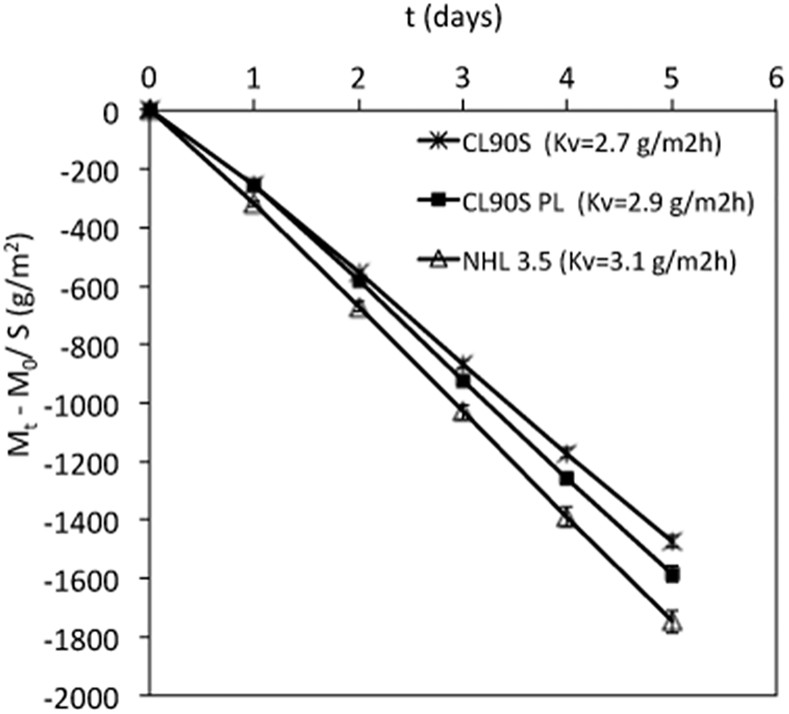
Water vapour permeability test. The weight variation per surface unit (M_t_—M_0_/S, g/m^2^) with error bars of the three hemp lime mixes is represented as function of time (t, days). K_v_ values (in g/m^2^h) are indicated in caption.

The water vapour permeability curves shown in Fig 3 indicate that the transpirability of hemp lime composites is also slightly influenced by the lime type. Among the three limes, the hydraulic one produces a composite with the highest water vapour permeability, although very similar values were found for the three mixes (K_v_ ~ 2.9 g/m^2^h). Moreover, the K_v_ values are similar to those measured in mortars made with dry hydrated lime and aggregate and designed for rendering applications (K_v_ between 2.6 and 3.1 g/m^2^h, depending on the binder-to-aggregate ratio [22]). This might indicate that the presence of hemp does not significantly improve the transpirability of the material, as expected. However, the mortars studied in Arizzi and Cultrone’s work [22] developed shrinkage fissures that are known to increase the water vapour diffusion within the mortar [27]. This suggests that the K_v_ values of the same mortars without shrinkage fissures should be lower than those of the lightweight hemp lime composites studied here, which did not suffer shrinkage.

### Water absorption and drying

**Fig 4:**
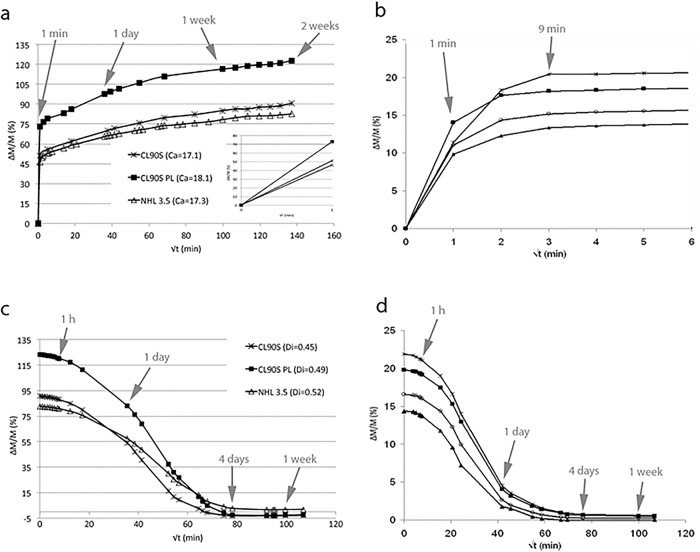
Free water absorption (a and b) and drying (c and d) tests. Curves in a and c represent the behaviour of the three hemp lime mixes whilst curves in b and d refer to a group of mortars made with aerial lime and stone aggregate and different binder-to-sand ratios (from Arizzi and Cultrone, 2014). The weight variation (ΔM/M, in %) of the three hemp lime mixes is represented as a function of the square root of time (t, min^-1/2^); the inset in (a) shows the first minute of the water absorption curves; the water absorption coefficient (C_a_, in g min^-1/2^) and drying index (D_i_) are indicated in the caption of Fig 4(a) and 4(c), respectively. Arrows indicate the time in minutes (min), hours (h), days or weeks.

During 2 weeks of immersion under water, the three hemp lime composites absorbed a large amount of water (ΔM/M ~ 120%, Fig 4a) compared to lime mortars made with a stone aggregate (ΔM/M ~ 15–20%, Fig 4b [22]). At the end of the 2 weeks, hemp lime samples started losing some shives and, at the same time, releasing some substances that changed the colour and density of water. To avoid wrongly interpreting the water sorption behaviour, the water absorption test was then stopped before samples reached saturation and the drying test was started.

As shown in Fig 4a and 4b, hemp lime composites showed similar and, at the same time, very different behaviour compared to lime-based mortars made with a stone aggregate. In fact, the hemp lime samples showed the greatest and fastest water uptake during the first few minutes, as mortars with lime and aggregate do. However, hemp lime samples absorbed a much larger amount of water in only the first minute (ΔM/M ~ 46–72%), as indicated by the C_a_ coefficient in caption of Fig 4a, than the latter usually do (ΔM/M ~ 10–15%, Fig 4b [22]). Moreover, after a few minutes, mortars with lime and aggregate reach mass saturation and do not absorb more water during the rest of the test (Fig 4b [22]). Hemp lime composites, on the contrary, continued absorbing water at a slower rate over the following 13 days (Fig 4a). When the test was stopped, after 2 weeks, the weight of hemp lime samples had increased by up to 122%. Considering that saturation was not achieved and knowing that the hemp shives used in this study have a sorption ability of 210–250% (according to the technical sheet for this material), it is reasonable to expect that hemp lime composites can absorb double the amount of water measured here. The amount of water absorbed by samples, however, was different according to the lime type, being the lowest in NHL 3.5 (ΔM/M = 82%) and CL90S (ΔM/M = 90%) samples and the highest in CL90S PL ones (ΔM/M = 122%). These values approach those found by Collet et al. [28] in hemp mortars and hemp lime renders, which showed an accessible porosity of ~65% and water saturation contents between 87 and 168%.

Considering that the slope of the curves mainly differ over the first minute of the test (inset in Fig 4a) whilst being very similar in the three mixes after this period (Fig 4a), it is likely that the very early water sorption is influenced by lime type, which is responsible for the differences in the final amount of water absorbed. The hemp, instead, influences the material absorption during the rest of the test and is responsible for the great amount of water absorbed by the three hemp lime composites.

After 2 weeks of water absorption test, samples were left to dry; the weight variations during the drying test are represented in Fig 4c. Samples were completely dried 4 days after the beginning of the test, which corresponds to the same drying period for mortars made with aerial lime and limestone aggregate (Fig 4d [22]). The hemp lime composites, however, dry slower in the first day, due to the much greater amount of water absorbed. Also the drying index values found for the hemp lime mixes made with aerial lime (CL90S and CL90S PL, average D_i_ ~ 0.47) are the same as those obtained in Arizzi and Cultrone’s study (average D_i_ ~ 0.49 [22]). These findings suggest that, during drying of hemp lime composites, the water is first released by the porous structure of the hemp shives to the capillary network of the lime-based matrix, which influences the speed of water loss over the duration of the test (i.e. smaller pores empty later than larger ones [29]). This is in agreement with Faure et al. [30] who observed, by means of NMR, that water progressively migrates from the hemp towards the binder (then, the matrix).

The fastest drying occurred in CL90S samples, followed by CL90S PL and NHL 3.5 ones. The hydraulic components in the latter mix, however, might have reacted with the water released by the hemp, thus modifying the drying rate. The fact that NHL 3.5 is the only mix type whose ΔM/M value did not drop below zero at the end of the drying test, as in the other mixes (Fig 4c), despite the loss of some shives during the test, may suggest the occurrence of hydraulic reactions (i.e. formation of hydrated phases from the reaction between calcium silicates and water) that have slightly increased the weight of samples and decreased the speed of water loss.

### Water capillary uptake

**Fig 5:**
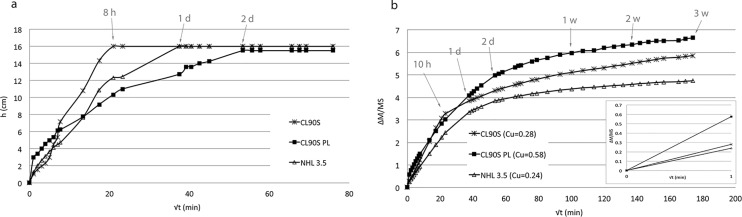
Capillary uptake test. In (a), the height variation (h, in cm) is represented as a function of the square root of time (t, min^-1/2^). In (b), the weight variation per surface area (ΔM/MS, in %) is shown as a function of the square of time (t, min^-1/2^); the inset shows the first minute of the capillary curves. The capillary coefficient (C_u_, in g min^-1/2^) of is indicated in the caption. Arrows indicate the time in hours (h), days or weeks.

The capillary uptake behaviour of the three hemp lime mixes is shown in Fig 5. Curves in Fig 5a represent the time taken by water to reach the top of the samples (visual saturation), which gives an idea of the degree of connectivity among pores present in the matrix. Curves in Fig 5b, instead, represent the capillary uptake rate and total amount of water absorbed (real saturation), which depends mostly on the total volume of pores accessible to water. The lag between visual and real saturation in the same sample indicates the presence of pores with different size (smaller pores are filled faster than bigger pores). Linking the information obtained in Fig 5a and 5b allows an assessment of the differences in pore volume, size and connectivity of the three hemp lime mixes.

Visual saturation was reached after only 8 hours (8 h) in CL90S samples, after 24 hours (1 d) in NHL 3.5 samples and after 48 hours (2 d) in CL90S PL samples (Fig 5a). The curves shown in Fig 5a are not linear for any of the three hemp lime mixes, which means that the water rise is not homogeneous over time. This is due to the fact that, during rise within the capillary pores of the matrix, water is at some point absorbed by the porous structure of the hemp shives. Since these pores are larger (up to 100 μm [10]) than the capillary pores present in the matrix of a lime mortar (most of them have pore radii between 0.1 and 1 μm [31]), a decrease in the water absorption speed is registered, as represented by the steps in the curves of Fig 5a. Therefore, knowing that the matrix of hemp lime composite is characterized by smaller pores than the hemp shives, and that these pores have a similar main size in the three mixes, it can be assumed that the time that water takes to reach the top of the samples mostly depends on the pore connectivity in the matrix. This might indicate that the matrix of CL90S mix is characterized by a higher degree of pore connectivity than CL90S PL. However, the latter composite is characterized by the highest volume of pores because, at the end of the test, CL90S PL samples showed the greatest and fastest water uptake, followed by CL90S and NHL 3.5 samples (see C_u_ values in caption of Fig 5b).

After 3 weeks of the capillary test, mass saturation was not achieved because the hemp shives continued absorbing water from the bottom of samples, as represented by the slower water uptake in the second part of the curves in Fig 5b. At the same time, due to exposure of the samples to air, water was released by other shives placed in the upper part of samples, thus inducing the occurrence of dry and wet zones in the same sample. As described by Lawrence et al. [32], the hemp—lime system never achieves a steady-state moisture content and this obviously affects the overall hygrothermal performance of hemp—lime mortars.

The capillary test was then stopped, because measurements could not be considered reliable after this period.

### Microbiological study of hemp shives and hemp lime composites

**Fig 6:**
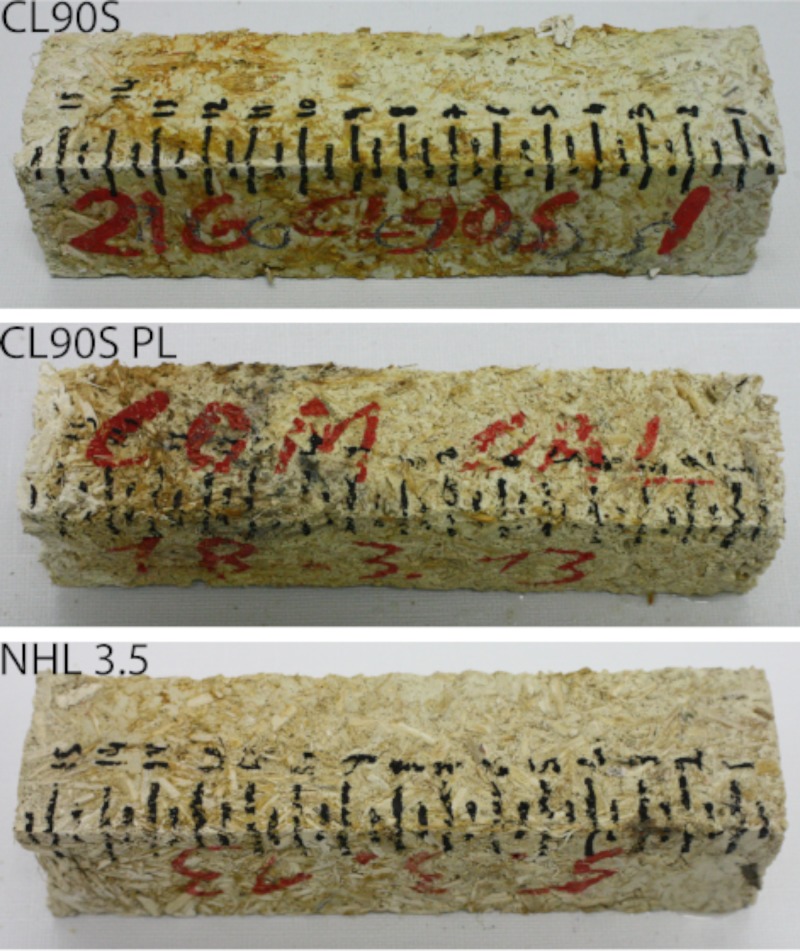
Appearance of the hemp lime composites after the capillary test. Orange and black stains are caused by the strong microbial attack induced by the presence of hemp and by dry-wet zones in samples.

During the capillary test all hemp lime mixes manifested new microbial growth (Fig 6), caused by the microbial community already present in the hemp shives and enhanced by the existence of dry and wet zones in the samples. The microbiological attack, in fact, was more intense in CL90S PL samples, due to the higher amount of water absorbed by the hemp shives (Figs 4 and 5b).

**Fig 7:**
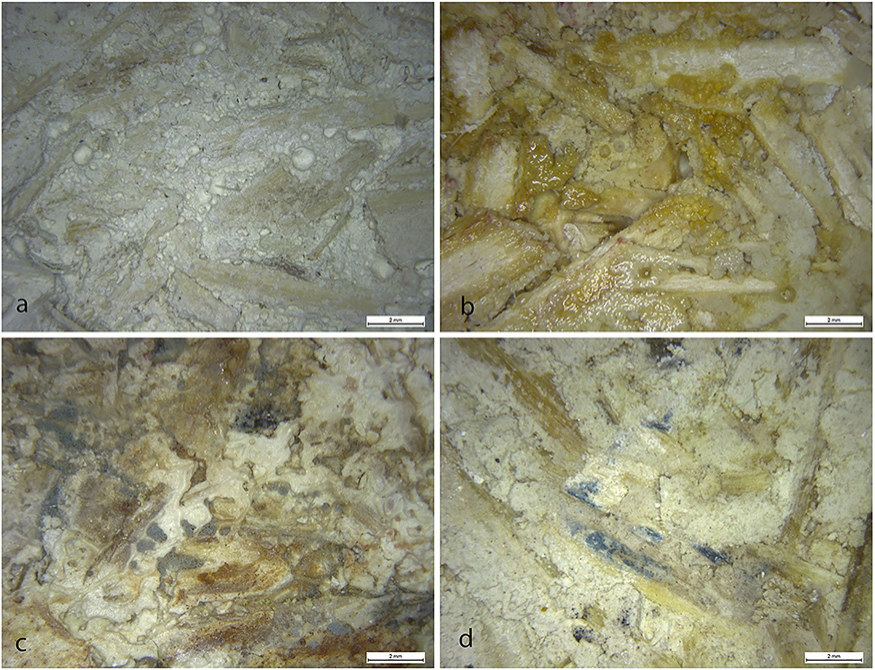
Surface of the hemp lime composites before (a) and after (b-d) the capillary test. The samples shown are: (a), CL90S; (b) CL90S; (c) CL90S PL; (d) NHL 3.5.

Fig 7 shows the appearance of the surface of the three hemp lime mixes observed by means of a video microscope, before (Fig 7a) and after (Fig 7b–7d) the capillary test. The attack of microorganisms is evident, due to the presence of orange (Fig 7b) and black (Fig 7c) stains all over the surface of sample and sometimes concentrated on the surface of the hemp shives (Fig 7d).

After the microbiological study carried out on the hemp shives, different types of microorganisms were isolated: four types of Gram-positive bacilli, one type of Gram-negative bacilli, one type of Gram-positive cocci, one type of actinobacteria and one type of fungi (Ascomycete).

**Fig 8:**
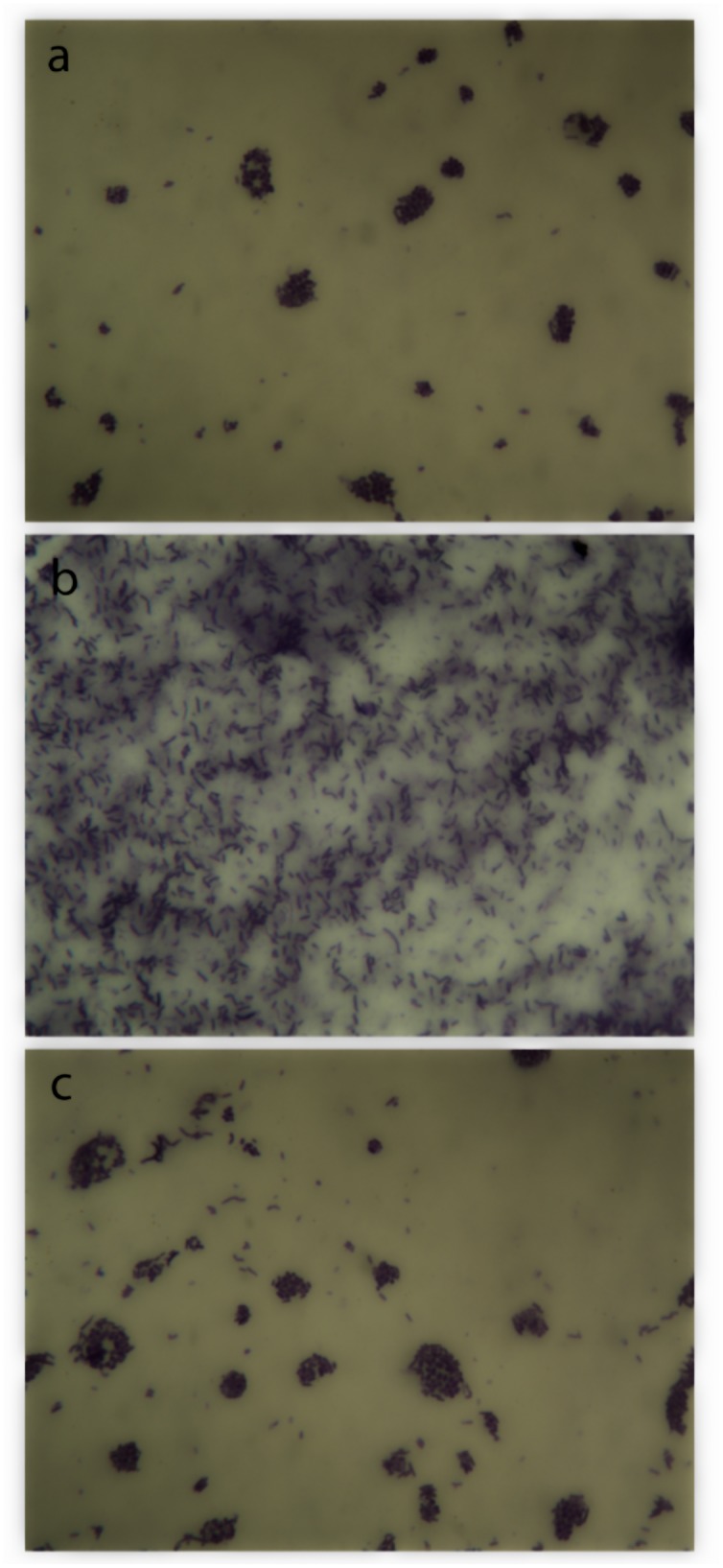
Microscopic images (1250X) of bacteria isolated from the hemp lime composites. (a), Gram positive sporulated bacillus; (b), Gram positive bacillus; (c), mix of both bacteria.

**Fig 9:**
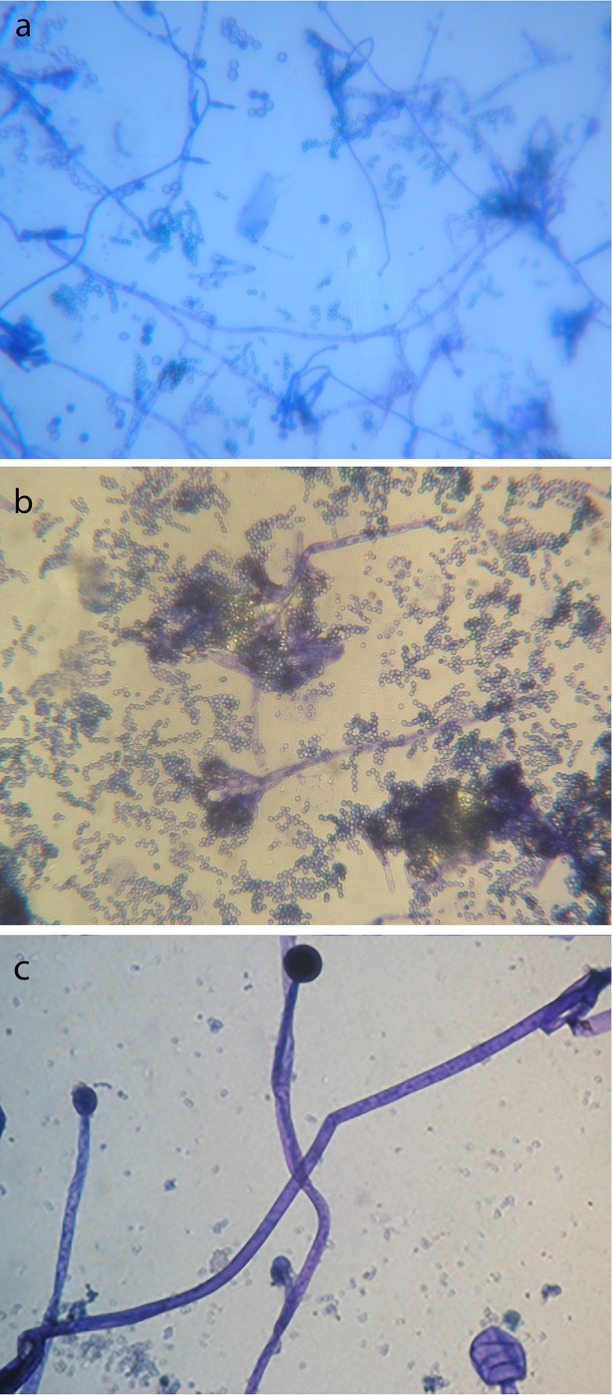
Microscopic images (750X) of fungi isolated from the hemp lime composites. (a), *Paecilomyces sp*; (b) *Penicillium sp* (c) *Rhizopus sp*.

In the mixes, however, only two types of bacteria (Gram-positive bacilli, Fig 8) were found, as they are the only alkaliphiles (i.e. class of extremophilic microbes able to survive in alkaline environments) among the microorganisms isolated in the hemp shives. This finding indicates that the presence of lime prevented the growth of the majority of the microorganisms present in hemp. Notwithstanding, we have also observed that mixing hemp with a lime binder does not ensure sufficient protection for bio-decay, as previously believed [18]. In fact, three different types of fungi (*Penicillium*, *Paecilomyces* and *Rhizopus*, Fig 9), not previously identified in the hemp shives, were also isolated in the hemp lime composites. The growth of these fungi is not surprising as they are very frequent in the air; it is then plausible that their spores deposited in samples where mycelia easily developed, thanks to the presence of organic matter (hemp shives, dirtiness and bacteria), moist conditions and absence of ventilation.

### Chromatic parameters and ANOVA

**Fig 10:**
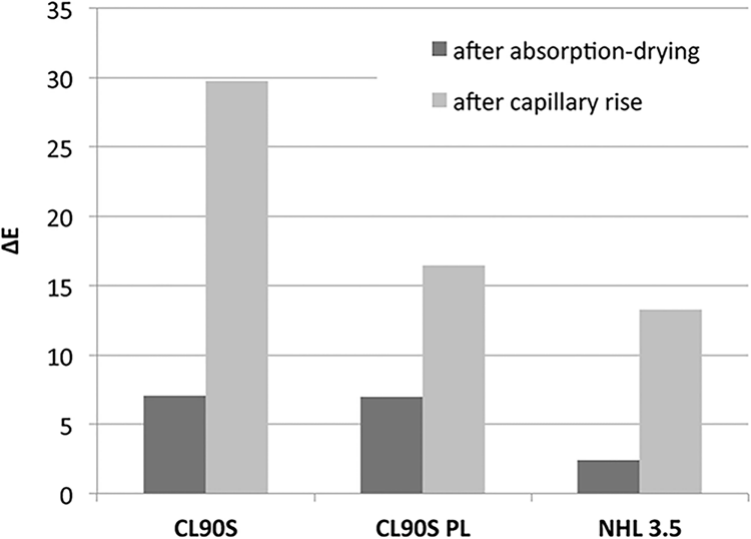
Chromatic changes in hemp lime composites. Overall colour difference values (ΔE) measured in the three mixes after the water absorption-drying and capillary uptake tests.

Variations in lightness (L*) and chromatic coordinates (a* and b*) of the three hemp lime mixes before and after the water absorption—drying and capillary tests are summarised in Fig 10 where the overall colour difference (ΔE) is represented. The results of ANOVA are summarised in Table 1; all the analyses passed the Shapiro-Wilk normality test (*p* is 0.647, 0.585 and 0.457 for L*, a* and b* values, respectively) and the equal variance test (*p* = 1). No statistically significant difference was found in the mean values of the three chromatic parameters among the different mixes (*p* is 0.578, 0.860 and 0.657 for L*, a* and b* values, respectively). Notwithstanding, some slight differences in the chromatic variations exist among the three hemp lime composites. As shown in Fig 10, the greatest ΔE value was registered in CL90S samples after the capillary uptake test whilst NHL 3.5 showed the slightest colour differences among the three mixes. According to ANOVA, no significant differences exist in a* and b* mean values among the two hygric tests (*p* is 0.085 and 0.066 for a* and b* values, respectively). However, a statistically significant difference was found in the mean L* values among the two types of test (*p* = 0.003). To isolate which group differs from the others a Student-Newman-Keuls multiple comparison procedure was used, investigating among the different mix and test types, as shown in Table 2. Again, no differences were found among the three hemp lime mixes as well as between before and after the water absorption-drying test (*p* > 0.050). Instead, the comparison between before and after the capillary test and between the two hygric tests resulted in a *p* < 0.050.

**Table 1:** ANOVA results for the chromatic parameters (L*, a* and b*) of the three hemp lime mixes after the hygric tests.

Dependent variable L*				
Source of variation	*df*	MS	*F*	*p*
**Mix**	2	4.128	0.630	0.578
**Test**	2	224.220	34.206	0.003
**Residual**	4	6.555		
**Total**	8	60.364		
Dependent variable a*				
**Source of variation**	***df***	**MS**	***F***	***p***
**Mix**	2	0.500	0.156	0.860
**Test**	2	15.494	4.842	0.085
**Residual**	4	3.200		
**Total**	8	5.599		
Dependent variable b*				
**Source of variation**	***df***	**MS**	***F***	***p***
**Mix**	2	7.602	0.467	0.657
**Test**	2	94.692	5.814	0.066
**Residual**	4	16.287		
**Total**	8	33.717		

*df* = degrees of freedom; MS = mean square variance; *F* = ratio of variance; *p* = significance

**Table 2:** Student-Newman-Keuls multiple comparison procedure on the L* parameter among the different hemp lime composites and hygric tests.

Comparison for factor: Mix	Difference of mean values	*p* < 0.050
**CL90S PL vs. NHL 3.5**	2.202	no
**CL90S PL vs. CL90S**	1.802	no
**CL90S vs. NHL 3.5**	0.400	no
**Comparison for factor: Test**	**Difference of mean values**	***p* < 0.050**
**Before vs. after capillary rise**	15.988	yes
**Before vs. after water absorption-drying**	2.292	no
**After water absorption-drying vs. after capillary rise**	13.696	yes

*p* = significance

Both the ΔE values and the ANOVA results indicate that the colour variation was more intense in samples subjected to the capillary test, due to the growth of fungi and bacteria, which produced black (Fig 7b, causing the decrease of L*) and orange (Fig 7c and 7d, causing the increase of b*) spots, respectively. The dark staining (known as microbial biofouling), which is a result of the high concentrations of melanin in the cell walls of black fungi [33], is certainly the main cause of the intense chromatic variations measured in samples after the capillary test. Although the statistical analysis did not find any significant variation in the chromatic parameters before and after the water absorption-drying test, spectrophotometry showed that this test also induced chromatic changes, even though they were less visible at first sight.

### Water absorption and drying of the hemp shives at the micro-scale

**Fig 11:**
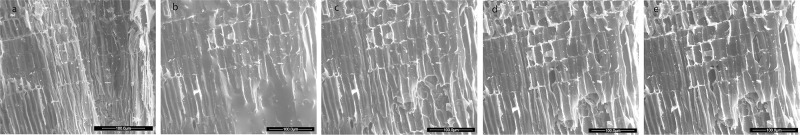
ESEM images of hydration and dehydration cycles performed on the hemp shives. (a) before hydration, at 3.5 torr corresponding to 70% of RH; (b) complete hydration, at 5.2 torr, corresponding to 100% of RH; (c) beginning of dehydration, at 4.6 torr, corresponding to 90% of RH; (d) dehydration at 4.1 torr, corresponding to 80% of RH; (e) end of dehydration, at 3.5 torr corresponding to 70% of RH. Observations were made at 2°C.

To better understand how hemp shives absorb and release water, a hemp shiv (about 5 mm long) was submitted to a cycle of hydration and dehydration in an environmental scanning electron microscope (ESEM, Fig 11). Fig 11a shows the hemp shiv before starting hydration and Fig 11b shows the swelling of the hemp shiv when soaked in water, as also described by Faure et al. [30]. During dehydration, the structure of the shiv starts to shrink due to the release of water (Fig 11c and 11d); at the end of dehydration, all the excess water absorbed by the shiv is lost and the conditions in the chamber are the same as at the beginning of the process (RH = 70%). However, the shiv does not recover the same volume as at the beginning (Fig 11e), indeed a certain swelling of the structure is still visible. It is worth taking into account that hydration-dehydration cycles were conducted over a short period of time, during which the shiv might not have had the time to recover its structure. However, our observations confirm that the absorption of water by the shiv induces a swelling of the structure that reduces the interfacial free energy and decreases the contact angle at increasing contact time of cellulose with water [11]. Moreover we demonstrated that it is possible that the shiv remains swollen after drying, because some water molecules remain linked by hydrogen bonds to cellulose, thus producing a change in its structure. As a consequence, the capacity of the shives to absorb water is increased and this would definitively influence its hygric behaviour over long periods in contact with water. This explains why hemp lime composites still absorb water 3 weeks after the beginning of the capillary test.

## Conclusions

The work reported in this paper aimed to compare the hygric behaviour and transpirability of sustainable building materials, made with hemp shives and three types of lime. It has been demonstrated that, although the three hemp lime mixes showed similar hygric performances, lime type influences the water transfer within this material. This indicates that the pore network of the matrix controls the water absorption and desorption rate and the differences in the amount of water absorbed. Hemp composites made with the natural hydraulic lime (NHL 3.5) presented higher transpirability and drying rate, lower water absorption by immersion and capillary uptake and less intense bio-deterioration compared to the aerial limes. This means that natural hydraulic lime has to be preferred in combination with hemp for the attainment of new and repair renders with good hygric performances. This choice also implies the development of a healthier environment in the interior of the building.

As expected, due to the presence of hemp shives, samples absorbed a much greater amount of water compared to a generic mortar made with lime and a stone aggregate. By studying the water absorption and desorption behaviour of a hemp shiv at the micro-scale, it has been demonstrated that the shiv is not able to recover its initial volume after the swelling caused by water. Indeed, even after drying, the shiv remains in a certain way swollen and this increases its capacity to absorb water. Although experimental conditions might play a role in the observed behaviour, this explains why the channels and pores constituting the shiv act as a nearly unlimited water reservoir.

However, the shives did not slow down the drying rate of samples and this is highly beneficial for a render, since it ensures the capacity to release water quickly when soaked, without allowing it to penetrate into the wall. Furthermore, the presence of hemp shives reduced to zero the shrinkage tension in all the mixes, indeed no fissures were observed.

During the hygric tests, strong microbial growth was observed in all the samples, caused by the presence of hemp shives and the occurrence of dry and wet zones in samples. Due to the high pH induced by the presence of lime, only alkaliphilic microbes already present in the hemp shives survived and grew when samples were in contact with water. However, other types of fungi, different from those isolated in the untreated hemp shives, were also identified. All these alkaliphilic microorganisms induced serious aesthetic damage to the hemp lime composites under wet-dry conditions. In all mixes, indeed, there was a variation of the chromatic values, due to an overall yellowing and a more localised dark staining. Although the microorganisms isolated are not pathogens, their growth still represents a serious problem for the use of hemp in building materials, therefore it needs to be reduced by taking the necessary preventive measures (e.g., chemical or physical disinfectant treatments of the hemp shives especially aimed at eliminating the alkaliphilic microbes).

Our study has demonstrated that although the ability of hemp to absorb large amounts of water is of benefit in relation to building hygrothermal performance, prolonged water absorption under particular environmental conditions (dry-wet cycles and scarce ventilation) may lead to bio-decay and this is certainly a disadvantage in building materials, especially those intended for repair works. Since the growth of microorganisms is strongly influenced by conditions of temperature and relative humidity, a future step in this research is to perform durability studies on these hemp lime composites, to assess their suitability as rendering materials under different climates.
